# Supplementary material for: 3D Model of the Early Melanoma Microenvironment Captures Macrophage Transition into a Tumor-Promoting Phenotype
Source: Cancers (Basel). 2021 Sep 12;13(18):4579. doi: 10.3390/cancers13184579 (PMC8471848; doi:10.3390/cancers13184579)
Supplement: Supplementary file 1 [file cancers-13-04579-s001.zip › cancers-1346878-supplementary.pdf]

Supplementary Materials: 3D Model of the Early Melanoma Microenvironment Captures Macrophage Transition to a Tumor-promoting Phenotype

Gabriela A. Pizzurro [1,†], Chang Liu [1,†], Kathryn Bridges [1], Amanda F. Alexander[1], Alice Huang[1], Janani Baskaran[1], Julie Ramseier[2], Marcus Bosenberg[2,3,4], Michael Mak[1]* and Kathryn Miller-Jensen [1,5]*

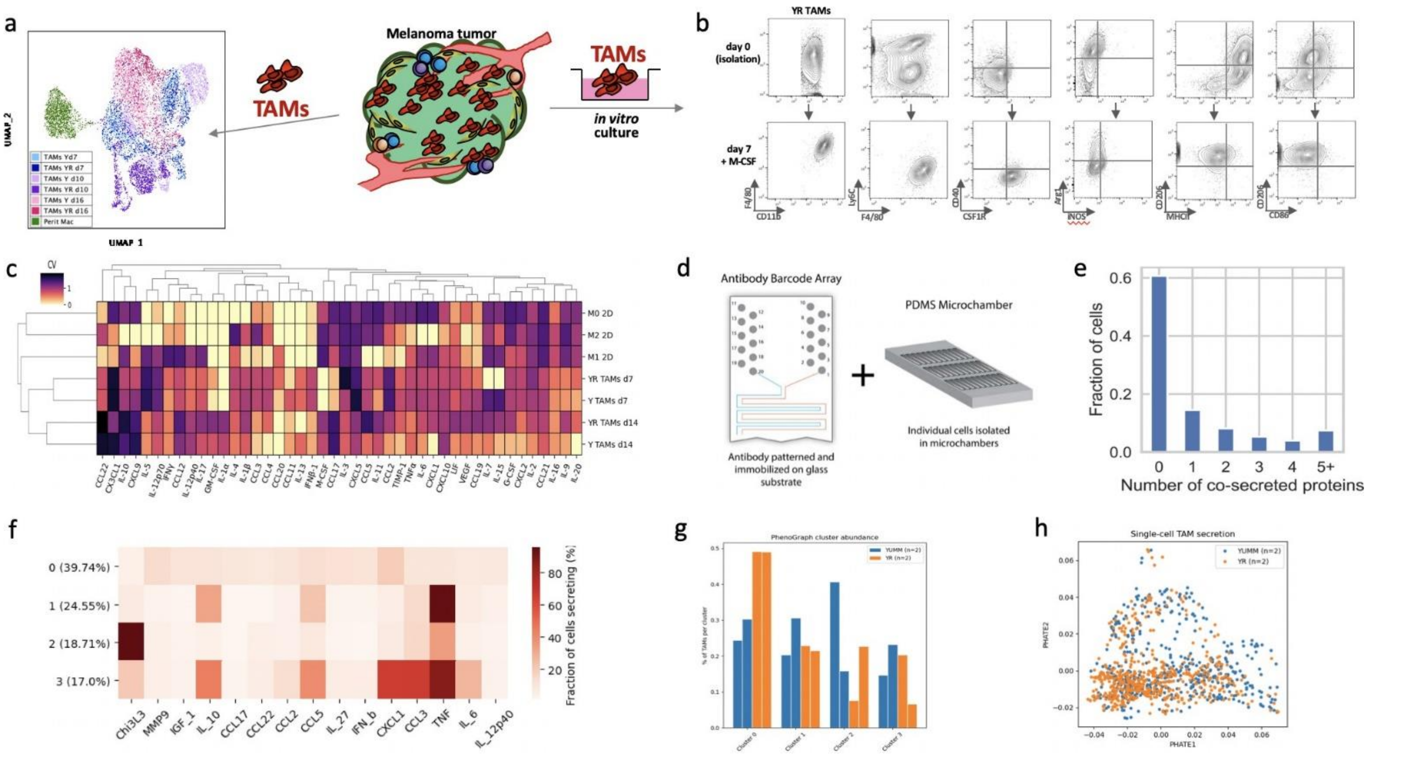

Figure S1. Characterization of the phenotype and functional profile of melanoma TAMs. **a**. We assessed melanoma TAM phenotype evolution by flow cytometry. Y and YR TAMs were processed at different timepoints (days 7, 10 and 16) and combined in a UMAP space for visualization. Peritoneal macrophage sample was added as a reference/control. **b**. Representative characterization of the phenotypic changes TAM undergo after isolation from the tumor and subsequent culture in in vitro 2D conditions for 7 days. **c**. Heatmap showing the variation of each measured protein across conditions. **d**. Single-cell barcode chip device components for detecting multiplexed individual cell secretion. **e**. Fraction of individual melanoma TAMs from d14 tumors secreting different amount of proteins, showing high percentage of quiescent cells. **f.** Phenograph clustering analysis of the single-cell secretion profile of Y and YR TAMs and classification into functional clusters. **g,h**. Analysis of single-cell TAM secretion and detected clusters, comparing Y and YR TAMs across different clusters.

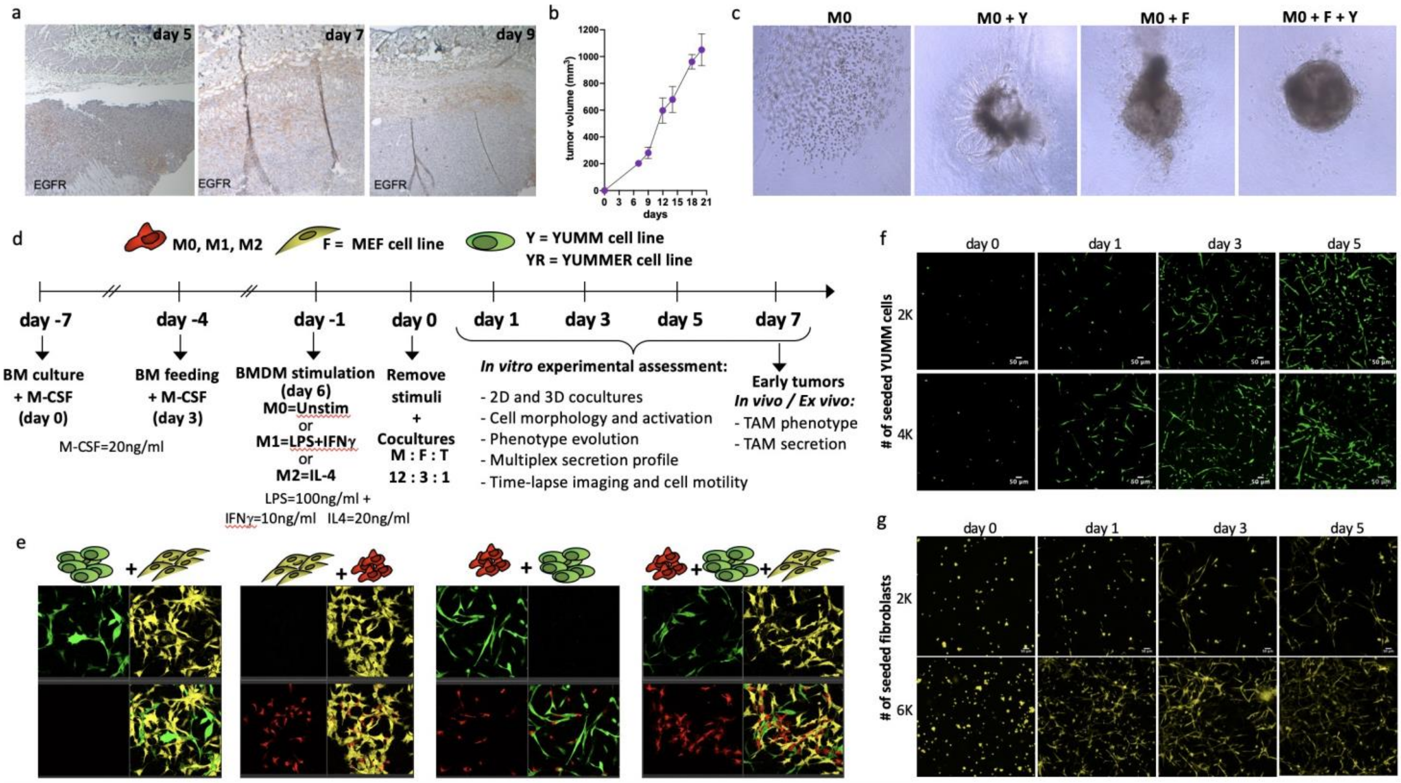

Figure S2. Mimicking the early melanoma TME with a 3D co-culture system. **a**. Rep- resentative images of fibroblast staining on YUMMER tumors. To locate and quantify fibroblasts in melanoma tumors at early time points, we performed immunohistochemistry staining for EGFR+ cells. After day 7, fibroblasts became segregated to the edge and this distribution was sustained over time. **b**. Growing curve, expressed in tumor volume (mm^3), of YUMM tumors, injected in B6 immunocompetent mice (n = 6). **c**. Represen- tative spheroids generated in Matrigel prior to embedding into collagen-I for further culture. **d**. Description of the timeline of the 3D coculture experiments. **e**. Representative confocal images of coculture combinations of the 3 components. Cocultures were pre- pared using single cell suspensions, with GFP+ YUMM cells, tdTomato+ 3T3-MEFs and Deep-red dye+ BMDMs. **f**. Timeline of 3D culture of GFP+ YUMM cells in collagen-I with different starting seeding cell density. **g**. Timeline of 3D culture of tdTomato+ 3T3MEF cells in collagen-I with different initial cell seeding densities.

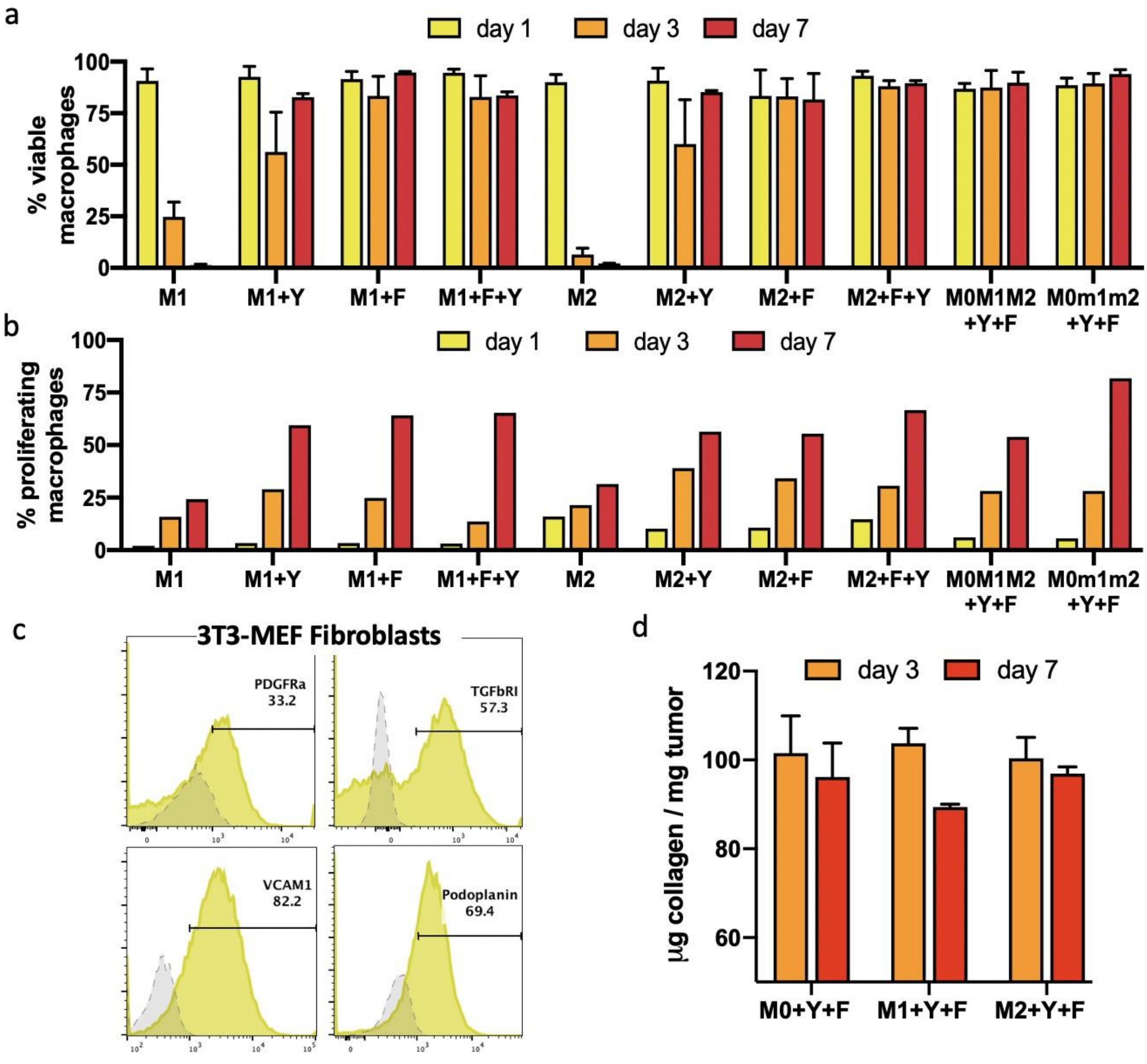

Figure S3. Rapid functional cell activation after early interactions in the 3D melanoma TME. **a**. Survival of polarized BMDMs in 3D cultures over time. Viabil- ity of macrophages was assessed after being cultured alone or in combination with the other cell components, at d1, d3 and d7 (n = 2). **b**. Proliferation of polarized BMDMs in 3D cultures overtime. We analyzed cell divisions of macrophages after CFSE staining, and considered in the proliferating fraction all cells that had undergone at least one division cycle. In M0M1M2, polarized BMDMs were mixed in equal parts in the same culture. In M0m1m2, polarized BMDMs were mixed in ratio 4:1:1 in the same culture. **c**. Representative histograms of FACS characterization of 3T3MEF fibroblast marker expression. **d**. Comparison of total soluble collagen-I in M+F+Y 3D cocultures with different initial polarization states at d3 and d7 (n = 4). * $p < 0.05$.

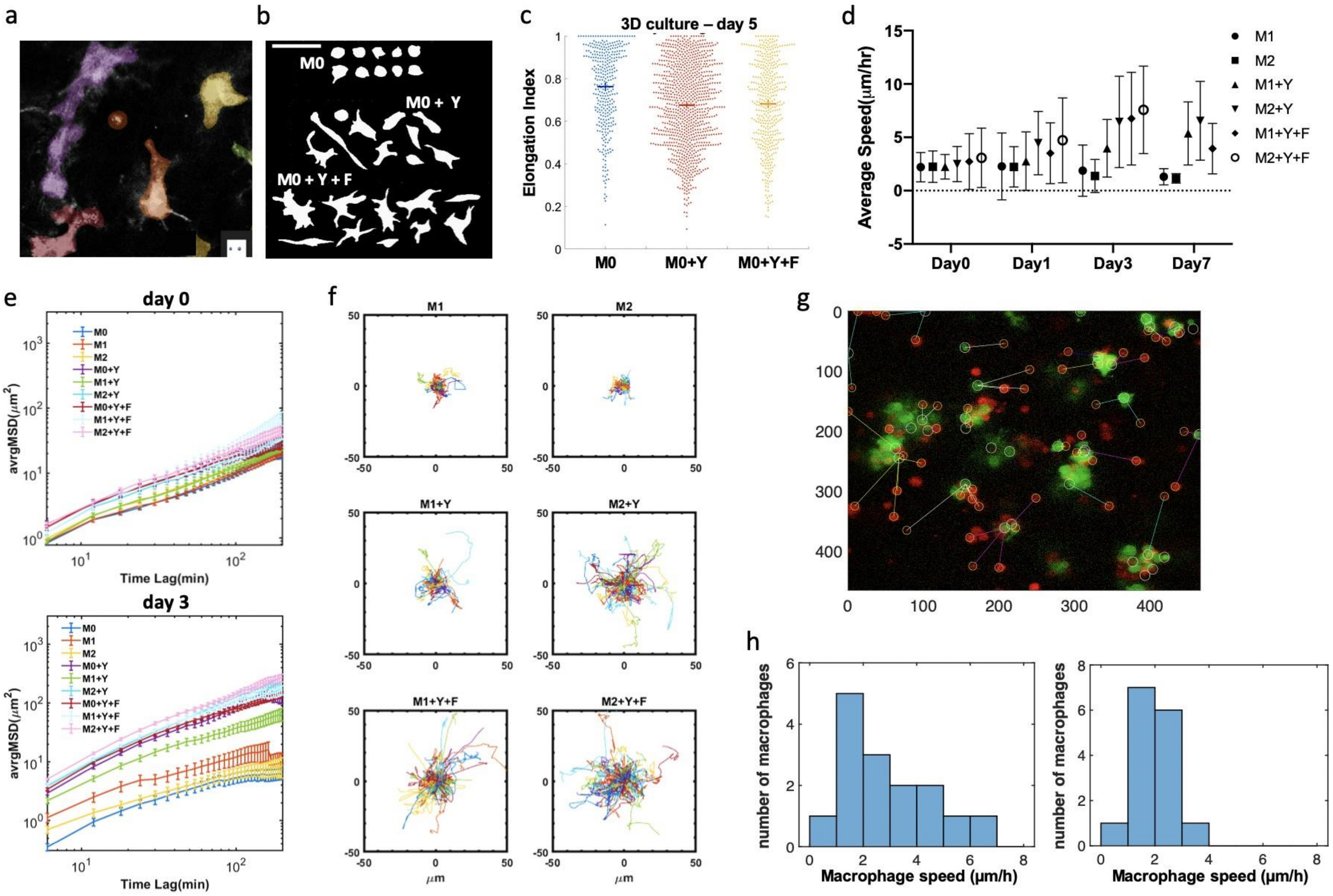

Figure S4. Significant morphology and motility changes in BMDM activation in 3D co-cultures over time. **a**. Representative image to demonstrate segmentation perfor- mance. **b**. A summary of representative macrophage morphology in each condition, 36h post gel embedding. Scale bar is 100 μm. **c**. Comparison of the elongation index of macrophages at day 5 in different 3D environments. The elongation index is defined as the width/length ratio of the maximum bounding box of macrophage. **d**. Average speed of M1 and M2 BMDM 3D cultures from day 0 to day 7. Data shown as Mean +/- SD. **e**. Average MSD of unstimulated and polarized BMDMs (M0, M1 and M2) at the initiation of the 3D cultures and at day3. Data shown as Mean +/- SEM. f. Overlaid migration trajectories of polarized BMDMs (M1 and M2) at day 3. **g**. Representative image of the initial stage of M0+Y 3D coculture used to identify macrophages and locate and calculate their distance in x,y,z to the closest tumor cell. **h**. Histograms of the average speed of M0 BMDMs either closest to (left) or farthest from (right) tumor cells.

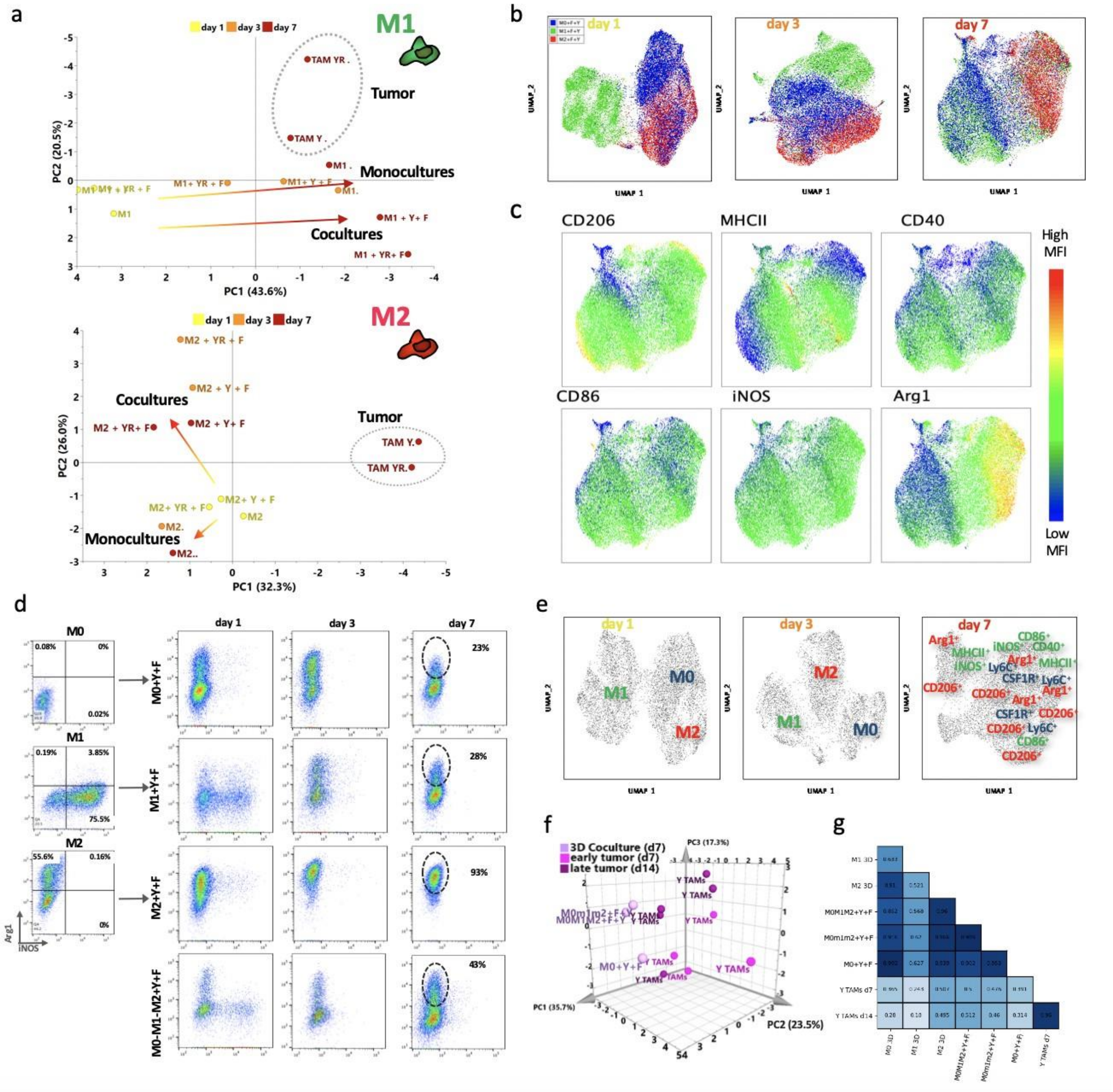

Figure S5. Evolution and modeling of BMDM phenotype into a TAM-like state. **a.** PCA analysis of pre-polarized macrophage phenotype trajectories over time in 3D cultures, compared to day 7 melanoma TAMs. **b**. UMAP clustering visualizing the plasticity and evolution of the macrophage phenotype in 3D cocultures, starting from different pre-polarized states (M0, M1 or M2). **c**. Heatmaps with details of the marker expression at day 7, starting from different pre-polarized states. **d**. Representative dot plots of M1/M2 markers iNOS/Arg1 in the pre-polarized BMDMs before coculture, and their evolution in the coculture with YUMM and fibroblasts. **e**. UMAP clustering visualizing the plasticity and evolution of the macrophage phenotype in 3D cocultures, starting from a mix of pre-polarized states (M0+M1+M2). At day 1 and day 3, sub- clusters were labeled according to their leading phenotype. At endpoint at day 7, the plot has superimposed annotations of the main markers expressed in different regions. **f**. PCA analysis comparing d7-coculture macrophage phenotype to d7 and d14 melanoma TAMs. **g**. Pearson correlation. In M0M1M2, polarized BMDMs were mixed in equal parts in the same culture. In M0m1m2, polarized BMDMs were mixed in ratio 4:1:1 in the same culture.

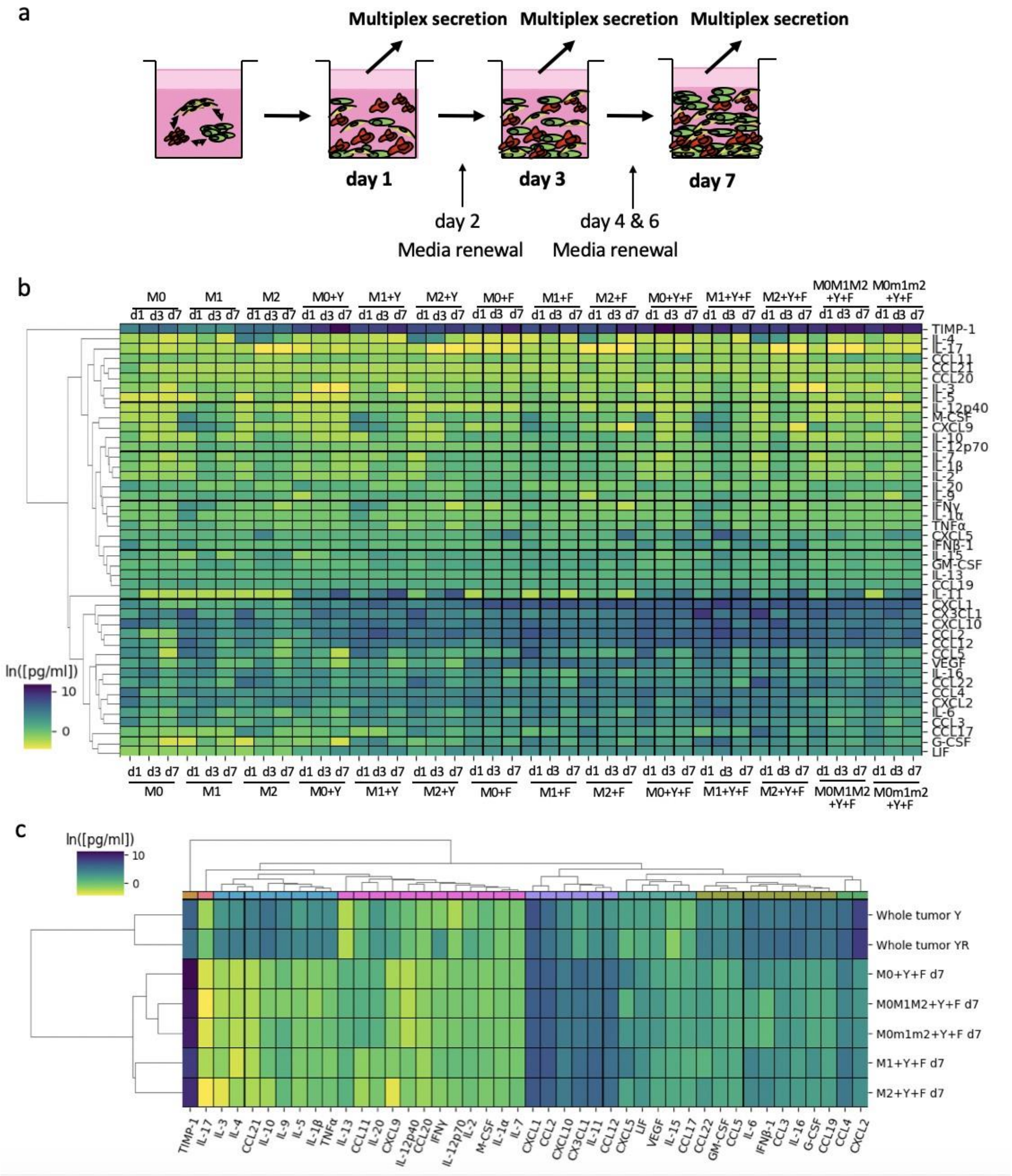

Figure S6. BMDM transition into an immunosuppressive TAM-like functional pro- file after 7 days of 3D co-culture. **a**. Schematic diagram of the collection of supernatants from the 3D cultures over time, M+Y+F in this example. **b**. Quantification of the condi- tioned media of 3D cultures a different timepoints. **c**. Hierarchical clustering of 43-plex secretion profile analysis of whole fraction of YUMM and YUMMER tumors at d7, compared to the different 3D cocultures at day 7. In M0M1M2, polarized BMDMs were mixed in equal parts in the same culture. In M0m1m2, polarized BMDMs were mixed in ratio 4:1:1 in the same culture.
